# Effects of cleaning spray use on eyes, airways, and ergonomic load

**DOI:** 10.1186/s12889-022-14954-4

**Published:** 2023-01-13

**Authors:** Karin Lovén, Anders Gudmundsson, Eva Assarsson, Monica Kåredal, Aneta Wierzbicka, Camilla Dahlqvist, Catarina Nordander, Yiyi Xu, Christina Isaxon

**Affiliations:** 1 Ergonomics and Aerosol Technology, Lund University, Lund, Sweden; 2 Occupational and Environmental Medicine, Lund University, Lund, Sweden; 3 Occupational and Environmental Medicine, University of Gothenburg, Gothenburg, Sweden

**Keywords:** Occupational exposure, Aerosol, Survey, Symptoms, PNIF, BUT, Physical workload

## Abstract

**Background:**

Cleaning workers are exposed to chemicals and high physical workload, commonly resulting in airway problems and pain. In this study the response in the upper airways and the physical workload following airborne and ergonomic exposure of cleaning spray was investigated.

**Methods:**

A survey was answered by professional cleaning workers to investigate their use of cleaning sprays and the perceived effects on eyes, airways and musculoskeletal pain. A human chamber exposure study was then conducted with 11 professional cleaning workers and 8 non-professional cleaning workers to investigate the airborne exposure, acute effects on eyes and airways, and physical load during cleaning with sprays, foam application and microfiber cloths premoistened with water. All cleaning products used were bleach, chlorine, and ammonia free. The medical assessment included eye and airway parameters, inflammatory markers in blood and nasal lavage, as well as technical recordings of the physical workload.

**Results:**

A high frequency of spray use (77%) was found among the 225 professional cleaning workers that answered the survey. Based on the survey, there was an eight times higher risk (*p* < 0.001) of self-experienced symptoms (including symptoms in the nose, eyes and throat, coughing or difficulty breathing) when they used sprays compared to when they cleaned with other methods. During the chamber study, when switching from spray to foam, the airborne particle and volatile organic compound (VOC) concentrations showed a decrease by 7 and 2.5 times, respectively. For the whole group, the peak nasal inspiratory flow decreased (-10.9 L/min, *p* = 0.01) during spray use compared to using only water-premoistened microfiber cloths. These effects were lower during foam use (-4.7 L/min, *p* = 0.19). The technical recordings showed a high physical workload regardless of cleaning with spray or with water.

**Conclusion:**

Switching from a spraying to a foaming nozzle decreases the exposure of both airborne particles and VOCs, and thereby reduces eye and airway effects, and does not increase the ergonomic load. If the use of cleaning products tested in this study, i.e. bleach, chlorine, and ammonia free, cannot be avoided, foam application is preferable to spray application to improve the occupational environment.

## Background

Cleaning workers, facing occupational exposure to cleaning products, constitute a large part of the workforce worldwide. At risk are also others in occupations involving cleaning tasks, e.g. hospital workers [1]. Several previous studies have shown that the use of cleaning products can cause adverse health effects, including skin, eye, and airway problems [2–6]. For example, Medina-Ramón et al. [7] showed through an extensive questionnaire study involving 4521 women that both former and currently employed female domestic cleaning workers had a higher prevalence of asthma than women who had never worked in cleaning. Furthermore, Lee et al. [8] performed a survey involving 183 cleaning workers, which showed that 31% reported respiratory symptoms and 15% reported eye symptoms at least monthly. Corresponding values for daily symptoms were 8% and 4%, respectively. It has been suggested that cleaning products containing irritants or disinfectants play an especially important role in cleaning-related asthma [9–12]. Matulonga et al. [13] showed that regular use of bleach for home cleaning is associated with developing adult-onset asthma and lower-airway symptoms.

Cleaning products are commonly applied by spraying. This practice generates an aerosol (i.e. airborne particles and gas phase compounds) and hence a higher risk of exposure than other application methods. A few studies have indicated that cleaning sprays increases the risk of new-onset asthma and other respiratory symptoms [8, 14–17]. Two studies using self-reported diary of exposure and symptoms, and self-recorded expiratory flow measurements have shown short-term effects among professional cleaning workers with asthma and/or chronic bronchitis [18, 19]. Medina-Ramón et al. [18] observed increases in daily reported lower respiratory tract symptoms when using sprays, but no significant change of upper respiratory tract symptoms or of peak expiratory flow (PEF). Further, Vizcaya et al. [19] showed that forced expiratory volume in one second (FEV_1_) and PEF decreased for asthmatic female cleaning workers (*n* = 21) during days with a high use of cleaning sprays. Svanes et al. [20] have also shown a long-term association between declining lung function for forced vital capacity (FVC) and FEV_1_ and the use of cleaning products, including sprays.

Although a number of epidemiological studies have linked eye and respiratory effects to the use of cleaning products, and some specifically to cleaning spray use, knowledge of how frequently cleaning sprays are used by professional cleaning workers is lacking. Controlled exposure studies are also needed to systematically investigate acute effects of cleaning spray exposure, and to compare different cleaning methods from a health perspective. However, to our best knowledge, no controlled human chamber exposure study has been conducted to correlate cleaning spray exposure generated by different cleaning methods with health effects in non-asthmatics.

Cleaning is not only associated with eye and airway problems; it also entails a high physical workload with strenuous postures, heavy lifting, and repetitive movements [21]. Cleaning workers often suffer from pain in the neck and upper extremities and run an increased risk of disability retirement [22]. Thus, ergonomics should be considered so that interventions to address one risk factor do not worsen another risk factor.

The aims of this study were to 1) assess the proportion of spray use and self-reported symptoms among professional cleaning workers, 2) characterize the airborne particle and volatile organic compound (VOC) exposures generated by different cleaning methods, and 3) to examine acute effects on eyes, airways and inflammatory systems from these exposures in controlled laboratory settings. An additional aim was 4) to investigate the physical workload during use of different cleaning methods.

## Methods

### Survey

A survey was developed specifically for this study, written in simplified Swedish to accommodate the large part of the workforce with native languages other than Swedish. It was distributed to professional cleaning workers of schools, offices, stores, and hotels (and, to a lesser extent, hospitals, and industrial premises) in Southern Sweden in 2016. The survey covered questions about how often, and which sprays were used, as well as about whether the workers experienced any health-related symptoms in the nose, eyes and throat, coughing or difficulty breathing during spray use or when not using spray. In addition, questions were asked about the workers’ experience of musculoskeletal pain as well as medical background questions (including allergies, physician-diagnosed asthma, and smoking). For the symptom questions, Visual Analogue Scales (VAS) (100 mm) were used with the extremes labelled as “never” (0) and “always” (100). In total, 300 professional cleaning workers from a total of ten cleaning companies were informed about the study. Written informed consent was obtained from all workers that chose to respond, and the study was approved by the regional ethical review board at Lund University, Sweden. The full survey as well as the implementation of the survey is described in more detail elsewhere (in Swedish) [23].

### Human chamber exposure

The study population in the human chamber exposures comprised 19 volunteer subjects. Inclusion criteria were: a) females, b) no current asthma diagnosis, c) non-smoker (for at least six months), d) age range 18–65 years, e) adequate Swedish language skills, and f) written informed consent and voluntary participation. Efforts were made that all subjects should be employed as professional cleaning workers (henceforth denoted “cleaning workers”), recruited among the participants of the survey. Due to recruitment difficulties among this population, 8 of the 19 subjects were recruited using advertising posters and were not professional cleaning workers (denoted “non-cleaning workers”). The principles of written informed consent in the current revision of the Declaration of Helsinki (2013) were implemented in the study. The study was approved by the regional ethical review board at Lund University, Sweden.

The test subjects were introduced to the chamber environment on a separate occasion, prior to study start, to minimize any effect of being unfamiliar with the environment. A pre-study examination described below was performed. A physician checked each subject’s medical history and conduced a physical examination. Spirometry with reversibility test (Bricanyl) was performed and a venous blood sample for Phadiatop allergy screening was obtained. Subjects with a current asthma diagnosis and/or with any regular (not anticonception) medication would have been excluded. No subjects were excluded on these grounds. Table 1 shows the characteristics of all subjects.

**Table 1:** Characteristics of the two groups of participants as determined by the pre-study examination

	All subjects (*N* = 19)	Cleaning workers (*N* = 11)	Non-cleaning workers (*N* = 8)
Age, years [median (min – max)]	34 (22–56)	41 (33–56)	24 (22–25)
Former smoker (N; %)	4 (21)	4 (36)	0 (0)
Never smoker (N; %)	15 (79)	7 (64)	8 (100)
Eye symptoms, at least 1 time/week in the last year (N; %)	3 (16)	1 (9)	2 (25)
Nasal symptoms, at least 1 time/week in the last year (N; %)	3 (16)	3 (27)	0
Dry cough, at least 1 time/week in the last year (N; %)	1 (9)	1 (9)	0
Chronic bronchitis (N; %)	1 (5)	1 (9)	0
Bronchial hyperreactivity – history (N; %)	3 (16)	1 (9)	2 (25)
Atopy – history (N; %)	2 (11)	2 (18)	0
Physician-diagnosed asthma during childhood (N; %)	1 (5)	0	1 (13)
Phadiatop positivity (N; %)	2 (11)	0	2 (25)
Spirometry before Bricanyl, FVC% (median; min–max)	90 (67–119)	84 (67–116)	102 (89–119)
Spirometry before Bricanyl, FEV1% (median; min–max)	92 (63–118)	84 (63–118)	102 (83–111)
Spirometry after Bricanyl, FVC% (median; min–max)	93 (68–118)	84 (68–118)	96 (69–112)
Spirometry after Bricanyl, FVE1% (median; min–max)	97 (65–120)	87 (65–120)	104 (87–113)

Cleaning products were selected for the human chamber exposures based on the observations from the survey. Three frequently used professional cleaning products from an internationally known brand were chosen (here denoted Window, Bathroom (normal), and Bathroom (acidic)). The specific physicochemical characteristics of the aerosol emissions from each of the cleaning products are described in detail in Lovén et al. [24]. During the current study, the total aerosol concentration, both particle and gas phase, was measured continuously. Table 2 lists the ingredients from the Material Safety Data Sheets (MSDS) of the chosen products. Note that only substances with a content over 1% are required to be included in the MSDS. None of the products contained bleach, chlorine, or ammonia. The two bathroom products were provided as concentrates and were manually diluted with water to the recommended concentrations (1%). The two different spray bottles provided for the two bathroom products had adjustable nozzles with no fixed positions. Based on that the spray mists would cover similar target surface areas, nozzle positions of 180° (for Bathroom (normal)) and 360° (for Bathroom (acidic)) from a closed nozzle position were chosen. A foaming nozzle (the same type for all three products) was used for the foam exposures (the different exposure scenarios are described below). This nozzle was also adjustable, and a position of 360° was chosen.

**Table 2:** Substances listed in the Material Safety Data Sheets (MSDS) for the tested products

Product	Substance^a^	CAS No.^b^	Content (% weight)	Hazard category^c^
Window	Isopropanol	67–63–0	5	F, Xi
Bathroom (normal)*	Alcohols, C9–11, ethoxylated	68,439–46–3	1–3	Xn
	Sodium lauryl ether sulfate	68,585–34–2	1–3	Xi
Bathroom (acidic)*	Citric acid monohydrate	5949–29–1	10–20	Xi
	a-D-Glucopyranoside, 2-ethylhexyl	125,590–73–0	1–3	Xi

Cleaning products marked with a star (*) were provided as concentrate and manually diluted to recommended concentrations (1%) before the study. These bottles also had adjustable nozzles

^a^Only substances with a content over 1% are required to be included in the MSDS

^b^Chemical Abstracts Service (CAS) registry number

^c^Hazard category: flammable (F), irritant (Xi), harmful (Xn)

The exposure chamber consists of a 21.6 m^3^ stainless steel chamber with a glass window and a floor area of 3 × 3 m, the approximate size of a hotel bathroom. The chamber was furnished with a toilet, sink, mirror, and shower (consisting of two tiled walls and two glass doors). A controlled flow of clean air was provided by a separate custom-built air-conditioning system maintained a temperature of 22.4 °C ± 0.7 °C, a relative humidity of 26.0% ± 3.5% (normal range for indoor winter time) and an air exchange rate of 0.9 h^−1^. The air supplied to the chamber by the conditioning system was filtered through an activated carbon filter (resulting in incoming air VOC concentrations of <0.1 ppm and ozone concentrations of <0.1 ppb). The air also flowed through an ultra-low penetration air (ULPA) filter (resulting in supply air particle concentrations of <100 particles cm^−3^ for particles <0.5 µm and <1 particle cm^−3^ for particles >0.5 µm). The chamber is described in detail by Isaxon et al. [25].

Particle number concentration and size distributions (in the size range 0.5–20 µm) were continuously measured using an Aerodynamic Particle Sizer (APS model 3321, TSI Inc., USA). A Condensation Particle Counter (CPC model 3010, TSI Inc., USA) was used to measure the particle number concentration in the size range >0.01 µm and a VelociCalc (model 9565-P, probe 986, TSI Inc., USA) was used to measure the total VOC gas phase concentration in the chamber. The time resolution of the APS, CPC, and VelociCalc were 5 s. An Ozone Analyzer (model 49i, Thermo Fisher Scientific, USA) was used to monitor the ozone concentration in the chamber with a time resolution of 1 s. In addition, a personal aerosol monitor SidePak (model AM510, TSI Inc., USA) was worn in a belt around the waist of the subjects to estimate the total particle mass concentration of particles in the size range 0.1–10 µm (PM_10_) in the breathing zone, with a time resolution of 10 s.

Three exposure scenarios were studied: *spray* – spraying the product onto the surfaces and wiping with pre-moistened microfiber cloths, *foam* – product application by foam onto the pre-moistened microfiber cloths and wiping with the cloths, and *water* – wiping only with pre-moistened microfiber cloths. It was randomly assigned to each subject what scenario to conduct on the first exposure day. The microfiber cloths were machine-washed and pre-moistened with water prior to each exposure day. All three cleaning products, denoted as Window, Bathroom (normal), and Bathroom (acidic), were used in scenarios *spray* and *foam*, the only difference between these two scenarios was the way the products were applied (either as spray or as foam).

The subjects were provided a protocol with instructions of how to use the cleaning equipment, specifically on the number of pulses used to apply the cleaning product in order to obtain comparable exposure levels. The same cleaning tasks were performed in all three exposure scenarios. The window and mirror in the chamber were cleaned with the Window product, the toilet and sink were cleaned with the Bathroom (normal) product, and the two tiled walls and two glass doors in the shower corner were cleaned with the Bathroom (acidic) product. During scenario *water*, the same cleaning tasks were performed without using any cleaning products.

**Fig. 1:**
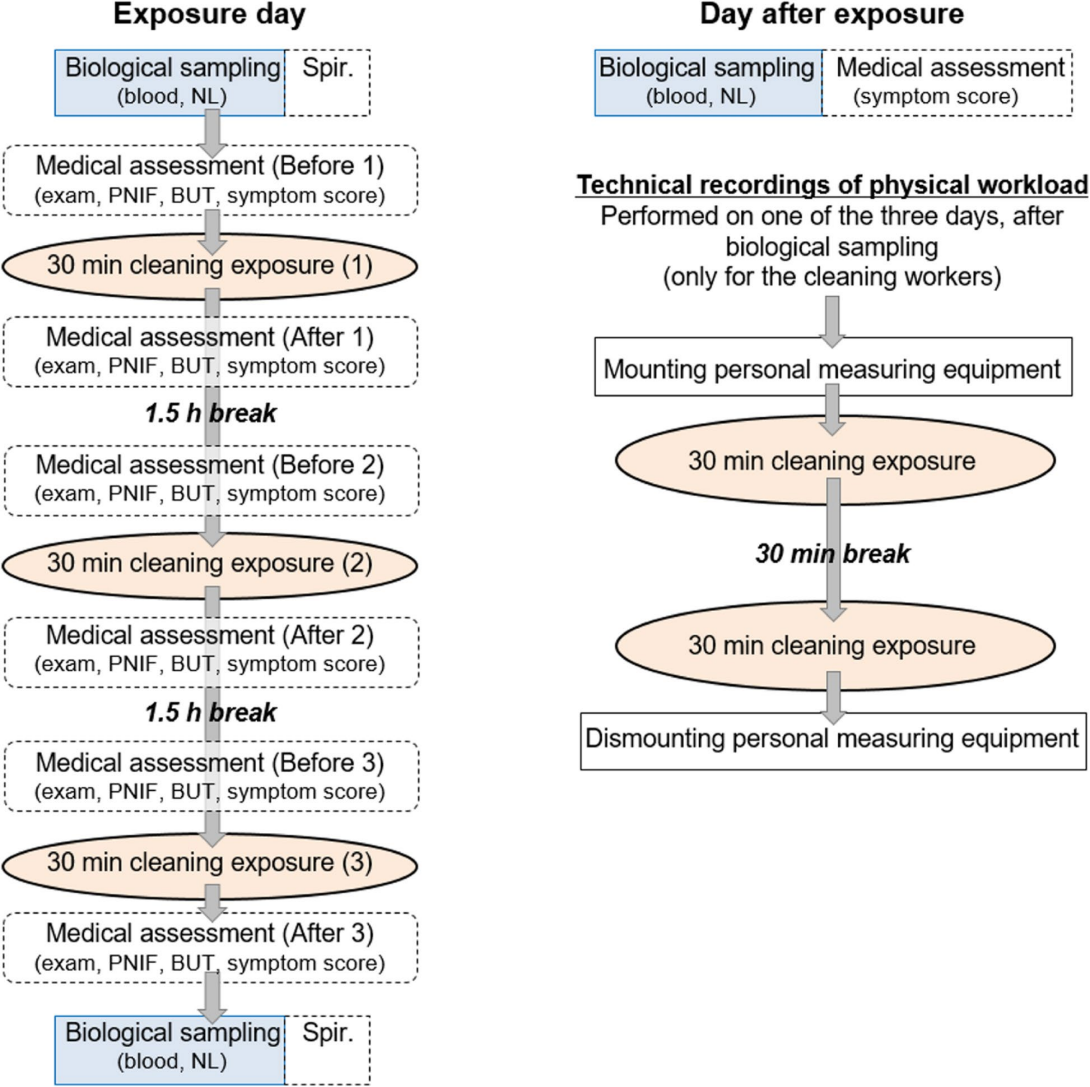
Flow chart of the study design for one exposure scenario. The different biological sampling and medical assessments included blood samples (blood), nasal lavage (NL) fluids, spirometry (spir.), physician-conducted examination (exam), peak nasal inspiratory flow (PNIF) measurements, measurements of the break-up time (BUT) of the tear film, and self-assessment symptom scores (symptom score)

Each subject performed one exposure scenario in one day (start ~ 8 am, end ~ 2 pm), for a total of three separate days with 1–3 weeks between scenarios. Two of the subjects only participated in two of the three exposure scenarios due to scheduling issues (one missed scenario *foam* and the other missed scenario *spray*). Figure 1 shows a flow chart of the study design for an exposure day. Each day included three 30-min cleaning exposures conducted inside the exposure chamber with a 1.5-h break between them. During the half-hour cleaning exposure, the bathroom inside the exposure chamber was cleaned eight times. Thus, during one exposure day each subject cleaned the bathroom 24 times, well in line with a normal number of bathrooms to clean daily for a hotel cleaning worker.

Biological sampling was performed twice a day, before and after the three exposures, shown in Fig. 1. Nasal lavage sampling was performed by flushing the nasal cavity with a room-temperature saline solution. The cells in the solution were immediately pelleted and the supernatant frozen at -80 °C [26]. The biological sampling procedure was repeated a third time, the morning after exposure, at ~ 8 am. Blood samples and nasal lavage samples were stored at -80 °C until analysis. The blood samples were analyzed for hemoglobin (Hb) as well as for neutrophils, eosinophils, basophils, lymphocytes, monocytes, total leukocytes and C-Reactive Protein (CRP) by standard protocol at Clinical Chemistry at Medical services, Region Skåne. Interleukin 6 and 8 (IL-6 and IL-8) in serum and nasal lavage fluid were analyzed by a multiplexed immunoassay Luminex method according to the manufacturer’s instructions (Bio-Rad Life Science, Hercules, USA). IL-6 and IL-8 were analyzed at the Division of Occupational and Environmental Medicine at Lund University.

Medical assessments were conducted directly before and directly after each half-hour cleaning exposure, a total of six times during an exposure day, as shown in Fig. 1. The physician examined the eyes (redness of conjunctiva, tears), the anterior nose (redness, secretion, blockage) and the throat (redness, secretion). Lung auscultation at normal and forced respiration was performed. Peak nasal inspiratory flow (PNIF) measurements to detect nasal obstruction were performed, as well as non-invasive assessments of the tear film stability by measuring the tear film break-up time (BUT). A PNIF meter (GM instruments, UK) was used together with a reusable mask. An ocular microscope (Keeler TearScope®, Keeler Instruments, UK) was used to assess the BUT [27, 28]. The measurements of PNIF and BUT were repeated three times during each medical assessment and an average value was calculated. Lung function testing, conducted by spirometry, was performed in the morning and the afternoon of the exposure day to record forced vital capacity (FVC) and forced expiratory volume in one second (FEV_1_) (Fig. 1). The spirometry was performed with SPIRARE 3 (Diagnostica, Norway) according to the European Respiratory Society [29] protocol. FVC and FEV_1_ were obtained and compared to reference material (ECSC/ERS 1993).

Subjects also filled in a short self-assessment symptom score with VAS scales during the medical assessments, based on those used by Dierschke et al. [30]. Symptoms from the eyes (itching, running, burning, sensation of dryness), the nose (itching, running, tingling, sensation of dryness, blocked), the pharynx (cough, sensation of dryness) and the lower airways (wheezing, shortness of breath, chest tightness) were registered. A question regarding strong smells was also included. The extremes of the VAS scales were labelled as “none” (0) and “a lot” (100). The symptom score was recorded by each subject six times during an exposure day and once in the following morning.

Heart rate and pulse were continuously monitored for the whole exposure day by a chest belt with a heart rate (HR) transmitter (model RS400, Polar Electronics, Finland). The subjects also wore a pulse watch, which recorded and stored the data.

Technical recordings of physical workload were performed on the eleven right-handed professional cleaning workers participating in the study. The physical workload during cleaning scenarios *spray* and *water* was assessed. To avoid aerosol exposure during the *spray* scenario, the cleaning spray bottle was filled with water instead of cleaning products, with minimal impact on the spraying performance for these recordings.

Postures and movements of the head, upper back and both upper arms were assessed by inclinometry [31]. Reference postures for upright head and back (0° inclination) and for vertical upper arms (0° elevation) were performed according to Dahlqvist et al. [32]. These references were later used to calculate work postures during scenario *spray* and *water*. Wrist postures and movements were recorded bilaterally with biaxial flexible electro-goniometers [33]. A reference posture (0° flexion/extension) was recorded according to Gremark Simonsen et al. [34]. The muscular load in the shoulder and forearm muscles was recorded using bipolar surface electromyography (EMG) [35]. The EMG signals were amplified, filtered (10–400 Hz) and sampled at 2048 Hz, and stored in a Mobi-8 data logger (TMS International, Oldenzaal, Netherlands). Further signal processing was then carried out as described in Nordander et al. [35].

The muscular load (electrical activity) recorded during work was normalized to the activity during maximal voluntary contractions (maximal voluntary electrical activity, MVE), and expressed as %MVE. The maximal voluntary contraction (MVC) for the shoulder muscles was recorded according to Nordander et al. [35], and for the forearms muscles according to Dahlqvist et al. [36]. Data were presented as group means of the 10^th^, 50^th^ and/or 90^th^ percentiles of the cumulative distributions of all recordings. Additionally, the recovery time (proportion of time <0.5% of MVE) was calculated as a percentage of the recorded time (% time) during scenario *spray* and *water*. To limit the amount of data, we chose to report data from the dominant (i.e. the right) side of the body.

### Statistical analysis

#### Survey

To examine the associations of specific symptoms (nose, eyes, throat, coughing, or difficulty breathing) with different cleaning habits, subjects with symptoms were defined as those who reported self-experienced symptoms “often” or “always” (defined as 51–75 and 76–100 mm, respectively, on the VAS scales), while “never”, “rarely” and “sometimes” (i.e. <51 mm on the VAS scales) were considered as showing no symptom. The notation “any symptoms” was defined as those who “often” or “always” reported one or more specific symptoms. Pearson’s chi-squared (χ^2^) test was used to compare symptom outcomes and spray/non-spray. Relative risk ratios (RRs) and their corresponding 95% confidence intervals (95% CIs) were calculated. The associations between self-experienced symptoms and age groups, smoking, allergies, and number of working year-groups were also analyzed using Pearson’s chi-squared (χ^2^) test. Five different age groups (<25, 26–35, 36–45, 46–55, >56 years) and five different number of working year-groups (<5, 6–10, 11–20, 21–30, >31 years) were defined for these analyses. Since a limited number of the respondents were male, all statistical analyses were performed for all workers without stratifying for gender. All analyses were performed with SPSS software (Statistics 24, IBM, USA).

#### Human chamber exposure

All the medical results were calculated as the changes from each subject’s baseline (i.e. individually normalized values). The baseline values were obtained in the morning of each exposure day (Before 1 in Fig. 1). A linear mixed model was used to analyze the differences in changes of outcomes at scenarios *spray* and *foam*, respectively, versus changes at scenario *water* for the PNIF and BUT measurements, and for the symptom scores. Age and individual baseline values were included in the model. The repeated covariance type chosen was autoregressive (AR(1)) since all the measurements at different times are autocorrelated. For all the other medical results and physical workload, the Wilcoxon signed rank test was used to analyze the difference between each exposure. All analyses were performed using SPSS software (Statistics version 22 and 24, IBM, USA).

## Results

### Survey

A total of 225 professional cleaning workers answered the survey constituting a response rate of 75%. The majority were female (73%). The participants had a median age of 44 (range 18–66) years and they had spent a median of 10 (range 1–40) years as a professional cleaning worker. In total 59 (26%) were current smokers, 11 (5%) had physician-diagnosed asthma, 17 (8%) had allergies as a child, and 41 (18%) had experienced allergic symptoms in adulthood. Cleaning sprays were used regularly by 174 out of the 225 respondents (77%). Table 3 shows the symptoms involving eyes and airways and in total (“any symptom”) in relation to the type of cleaning for all investigated cleaning workers, separated by gender.

**Table 3:** Distribution of self-experienced symptoms in the nose, eyes and throat, coughing, and difficulty breathing

**Symptom**	1: Spray users (*N* = 174)						2: Never spray users (*N* = 51)		
	a: During spray use			b: During cleaning other than with spray			During cleaning other than with spray		
	All N (%)	Female^a^ *N* = 128 N (%)	Male^a^ *N* = 38 N (%)	All N (%)	Female^a^ *N* = 128 N (%)	Male^a^ *N* = 38 N (%)	All N (%)	Female^a^ *N* = 37 N (%)	Male^a^ *N* = 8 N (%)
Any symptom	48 (28)	31 (24)	15 (39)	6 (3)	4 (3)	2 (5)	6 (12)	5 (14)	1 (13)
Eyes	14 (8)	11 (9)	3 (8)	2 (1)	1 (1)	1 (3)	3 (6)	3 (8)	0
Nose	34 (20)	23 (18)	10 (26)	5 (3)	4 (3)	1 (3)	2 (4)	2 (5)	0
Throat	26 (15)	19 (15)	7 (18)	4 (2)	2 (2)	2 (5)	6 (12)	5 (14)	1 (13)
Cough	18 (10)	10 (8)	7 (18)	2 (1)	0	2 (5)	1 (2)	1 (3)	0
Difficulty breathing	8 (5)	4 (3)	3 (8)	1 (1)	0	1 (3)	1 (2)	1 (3)	0

The number of people with symptoms at a frequency of “often” or “always” for (1.a) spray users during spray use, and (1.b) spray users during cleaning other than with spray, as well as for (2) never spray users during cleaning other than with spray, are shown in separate columns. “Any symptoms” is defined as those who “often” or “always” reported one or more specific symptoms. The different symptoms related to “Eyes”, “Nose” etc. refers to the sum of all various symptoms reported for eyes, nose etc.

^a^As not everyone provided gender information, the total number of answers is different from the sum of the gendered results

Cleaning workers who regularly use cleaning sprays (Table 3, column 1) have a significantly increased relative risk (RR) of experiencing “any symptoms” (RR = 8.0, 95% CI 3.5–18.2, *p* < 0.001) when using spray (column 1.a) compared to when cleaning with other methods (column 1.b). Furthermore, the group of cleaning workers who regularly use cleaning sprays (column 1) have a significantly increased risk of experiencing “any symptoms” (RR = 2.3, 95% CI 1.1–5.2, *p* = 0.02) during spray use (column 1.a) compared to the group who never used cleaning sprays (column 2).

Workers who use cleaning sprays more than five times daily (*N* = 36) have a significantly increased risk of experiencing one or more of the symptoms listed in the survey (RR = 5.0, 95% CI 1.3–19.5, *p* = 0.004) compared to the workers who use sprays 1–5 times per week (*N* = 29).

Smoking, allergies, age, or number of years as a cleaning worker had no significant influence on symptoms among workers who regularly use spray (*p* > 0.08 for all).

Of the professional cleaning workers, 174 (77%) stated that they experienced pain. The three most common locations were shoulders (49%), neck (45%), and hands and wrists (36%). The majority of the workers using sprays regularly (68%) answered that they experienced the same pain regardless of whether sprays were used or not. Few workers (9%) experienced more pain during spray use and only 5% experienced less pain.

### Human chamber exposure study

#### Aerosol concentrations

**Fig. 2:**
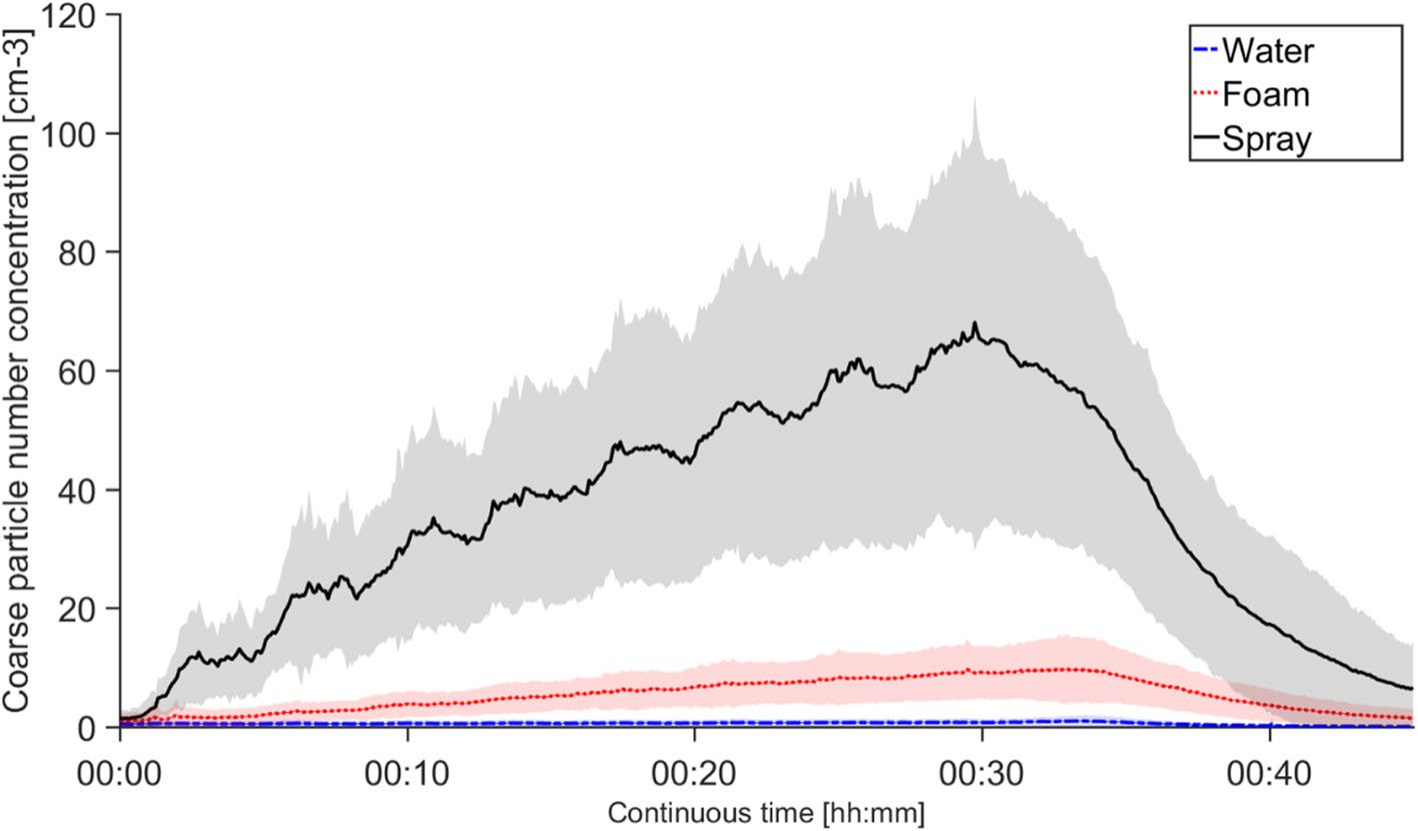
Number concentration of particles 0.5–20 µm generated during the three different cleaning scenarios. The concentration was measured using the APS and the average values for all 19 subjects are shown as the bold lines. The shaded areas are the standard deviation. Exposures lasted 30 min (from 00:00 to 00:30). After exposure the subject left the chamber, and the particles were vented through express ventilation (AER of ~10 h^−1^). Concentrations were not normalized to the individual amount of liquid used

An about 60-fold increase in number concentration of particles 0.5–20 µm, measured by APS, were seen during scenario *spray* compared to scenario *water*, for all subjects, and at the end of the 30-min exposure a maximum particle concentration with an average of 67 cm^−3^ was obtained (denoted as “average of maximum”) (Fig. 2). Using a foaming nozzle resulted in a 7 times lower particle concentration (average of maximum 9 cm^−3^). The professional cleaning workers and non-cleaning workers generated approximately the same average of maximum particle concentrations, 68 and 65 cm^−3^, respectively, in scenario *spray* and 8 and 13 cm^−3^, respectively, in scenario *foam*. Additionally, the standard deviation for the whole group reflects a large difference between individual users, irrespective of being cleaning workers or non-cleaning workers, both during spray and foam use (similar relative standard deviations, 52 and 57% respectively). Even when normalizing the particle number concentration to the individual amount of liquid used (average 414 g, range 198–486 for *spray* and average 461 g, range 278–533 for *foam*), there was no significant difference between the relative variation of concentration of particles 0.5–20 µm generated from spray and from foam use. The eight peaks in Fig. 2, most clearly seen in the standard deviation for *spray*, show the start of each of the eight bathroom-cleaning cycles. As in previous measurements of cleaning spray particles, including sprays used in this study [24], the particles generated during the exposure study had an average particle size around 1 µm.

**Fig. 3:**
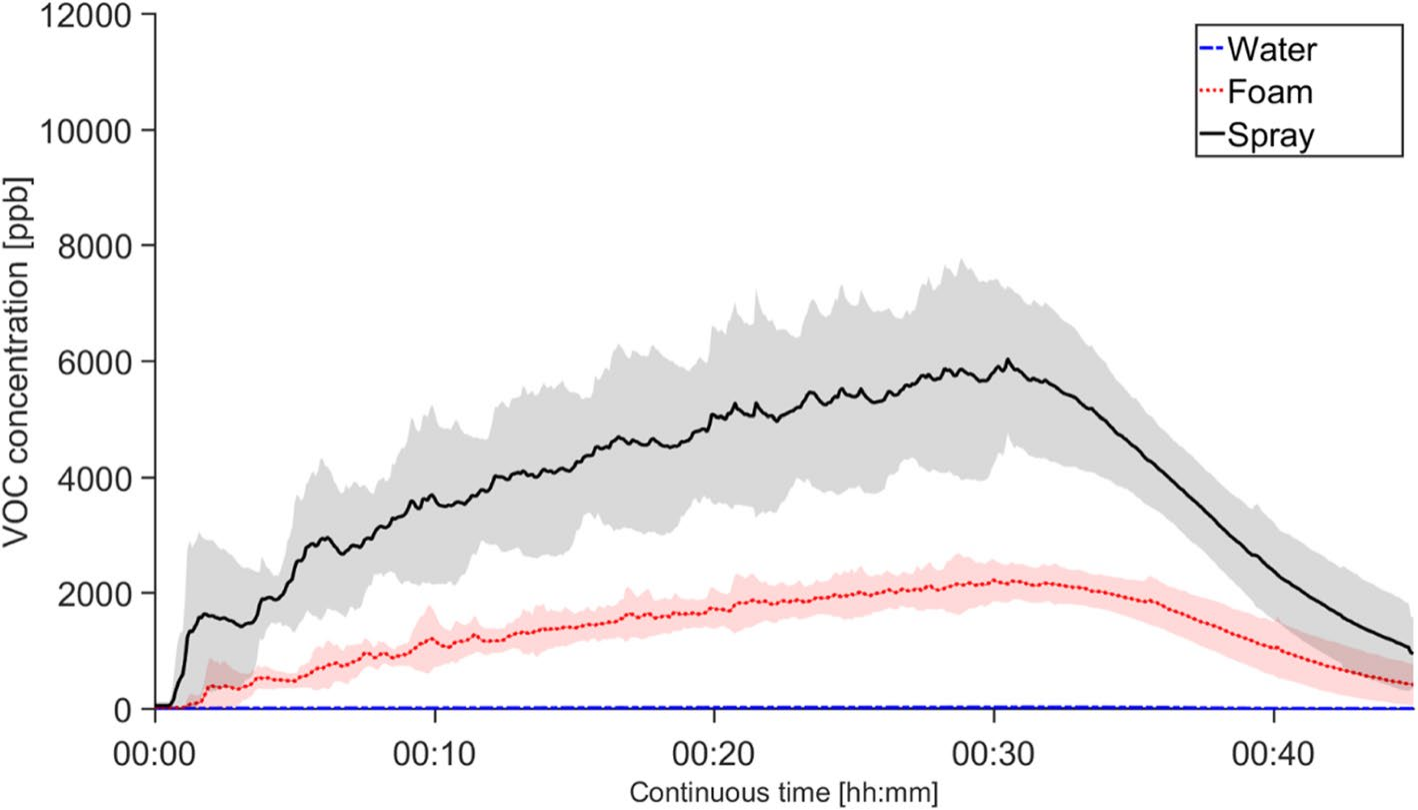
VOC concentration generated during the three different cleaning scenarios. The concentration was measured using the VelociCalc and the average values for all 19 subjects are shown as the bold lines. The shaded areas are the standard deviation. Exposures lasted 30 min (from 00:00 to 00:30). After exposure the subject left the chamber, and the VOCs were vented through express ventilation (AER of ~10 h^−1^). Concentrations were not normalized to the individual amount of used liquid

As with the particle number concentration measured by the APS, an about 60-fold increase in VOC gas phase concentration can be seen during spray use compared to the water exposure, for all subjects, with an average of maximum of 5550 ppb (Fig. 3). Using the foaming nozzle instead of spray resulted in a 2.5 times lower VOC concentration. This decrease (to an average of maximum of 2140 ppb) is not as large a decrease as that of the particle concentration. The cleaning workers and non-cleaning workers generated an average of maximum VOC concentrations of 6490 and 4070 ppb, respectively, in *spray*, and 2330 and 1840 ppb, respectively, in *foam*. Additionally, the standard deviation for the whole group again reflects individual differences during both spray and foam use, with relative standard deviations of 29 and 23% respectively.

**Fig. 4:**
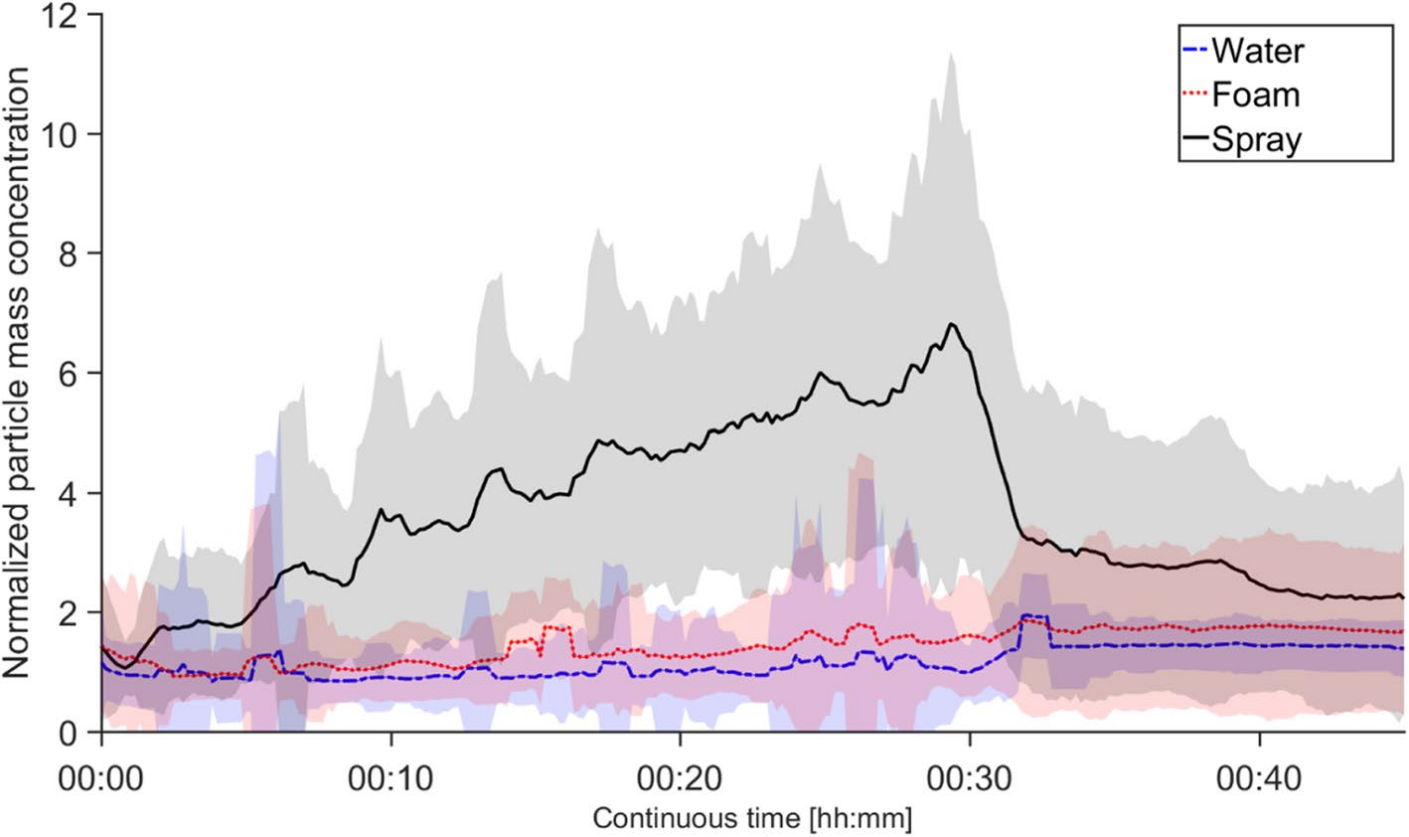
Total particle mass concentration generated during the three different cleaning scenarios. The concentration in the breathing zone of the subject was measured using the SidePak. The concentration was normalized by the average value of the *water* exposure from the APS data and calculated as a running one minute average, for all 19 subjects, to reduce noise due to instrument movement during the measurements. The shaded areas are the standard deviation. Exposures lasted 30 min (from 00:00 to 00:30). After exposure the subject left the chamber. Thereafter the SidePak was measuring the air outside the chamber

Total particle number concentrations (>0.01 µm) measured with the CPC was generally low (<500 cm^−3^) and did not show any significant differences between the different cleaning scenarios (not presented here). The total PM_10_ particle mass concentration in the breathing zone of the subjects measured by SidePak were about six times higher (average of maximum) during spray use than water use (Fig. 4), which is similar to the general particle concentration in the chamber shown in Fig. 2. However, no difference in total PM_10_ particle mass concentration was observed between the *foam* and the *water* exposures. All breathing zone concentrations were normalized using the average concentration of the *water* exposure from the APS data. The concentration was calculated as a running one-minute average for all 19 subjects. The ozone concentration was below the limit of detection (<0.1 ppb) throughout the study.

#### Self-assessed symptoms, medical assessments and biochemical analyses

Scores of the self-assessed symptoms for the whole group are shown in Table 4. The reported symptoms by subjects were limited, an increase in nasal symptoms (itching, running, tingling, sensation of dryness, blockage) could however be observed during the *spray* exposure for 50^th^ and 75^th^ percentiles compared to *water* (Table 4). Additionally, nasal symptoms increased in some of the subjects immediately after each half-hour exposure compared to directly before, which indicates that these subjects are affected by each individual exposure and not just by one whole day of cleaning. For scenario *foam*, only a minor increase in self-assessed nasal symptoms could be observed compared to *water*. For eye symptoms only a small increase for *spray*, for 75^th^ percentile, was observed and no changes at all for throat symptoms.

**Table 4:** Self-assessed symptoms during the three different exposure scenarios

	Time point	Water			Foam			Spray		
		Percentiles								
		25^th^	50^th^	75^th^	25^th^	50^th^	75^th^	25^th^	50^th^	75^th^
Nasal symptoms	Before 1	0	3	10	0	1	13	0	2	12.5
	After 1	0	0	0	0	0	9.3	0	10.5	18.5
	Before 2	0	0	7	0	0	10.3	0	0	9
	After 2	0	0	6	0	0	9	0	7.5	25.5
	Before 3	0	0	6	0	0.5	8	0	5.5	17.8
	After 3	0	0	4.3	0	3	10	0	11	19.8
	Day after	0	0	6	0	3	14.5	0	4.5	13.5
Eye symptoms	Before 1	0	0	5	0	0	0.8	0	0	8.3
	After 1	0	0	0	0	0	1.5	0	0	2.5
	Before 2	0	0	0	0	0	0	0	0	0
	After 2	0	0	4	0	0	7	0	0	1.3
	Before 3	0	0	0	0	0	0.5	0	0	5
	After 3	0	0	1.5	0	0	3	0	0	8.5
	Day after	0	0	0	0	0	2.5	0	0	0.8
Throat symptoms	Before 1	0	0	4	0	0	4.8	0	0	4.5
	After 1	0	0	6	0	0	7	0	0	9.5
	Before 2	0	0	6	0	0	0	0	0	0
	After 2	0	0	5	0	0	5.5	0	0	7.5
	Before 3	0	0	5.3	0	0	0.8	0	0	0
	After 3	0	0	7.8	0	2.5	8.3	0	0	11
	Day after	0	0	1.3	0	0	3.5	0	0	0

Values within the group of all subjects are presented as the 25th-, 50th- and 75th-percentiles from the summed results of the four different eye symptoms (itching, running, burning, sensation of dryness), five different nasal symptoms (itching, running, tingling, sensation of dryness, blocked), and two different throat symptoms (cough, sensation of dryness), using a visual analogue scale ranging from 0 to 100 mm

The measured values of BUT, PNIF, FVC and FEV_1_ for the whole group are presented in Table 5. The BUT 75^th^ percentile values decrease with an increased exposure for both foam and spray. An increase of PNIF values (25, 50 and 75^th^ percentile) throughout the day can be seen during *foam* and *water* (and in most cases higher values after each half-hour exposure), while this increase cannot be seen during *spray*. No significant changes could be observed for the spirometry values.

**Table 5:** Medical assessment using BUT, PNIF, and spirometry measurements during the three different exposure scenarios

	Time point	Water			Foam			Spray		
		Percentile								
		25^th^	50^th^	75^th^	25^th^	50^th^	75^th^	25^th^	50^th^	75^th^
BUT (sec)	Before 1	8.1	13.8	16.3	9.0	11.7	26.2	8.4	15.0	22.4
	After 1	7.9	11.4	15.9	8.1	11.7	14.4	8.8	12.1	17.1
	Before 2	10.1	13.7	22.1	9.7	11.6	15.0	8.0	11.8	17.8
	After 2	9.6	11.7	20.7	9.2	10.3	14.5	9.4	10.9	16.2
	Before 3	8.9	11.4	16.9	8.7	10.7	14.6	9.3	11.9	15.1
	After 3	7.9	13.8	20.7	9.5	11.5	14.1	8.7	11.5	16.1
PNIF (L/min)	Before 1	92	123	143	80	111	162	88	120	158
	After 1	97	127	170	88	105	158	99	108	141
	Before 2	97	127	155	79	115	144	90	117	160
	After 2	107	125	170	87	128	190	81	113	159
	Before 3	85	136	172	83	128	167	89	108	163
	After 3	101	133	183	97	120	179	88	120	158
Spirometry FVC (L)	Before 1	3.1	3.7	3.9	2.7	3.5	4.1	3.0	3.5	4.1
	After 3	2.8	3.5	4.1	2.9	3.5	3.8	2.9	3.6	4.1
Spirometry FEV_1_ (L)	Before 1	2.5	2.9	3.5	2.3	2.9	3.6	2.3	2.9	3.5
	After 3	2.3	3.1	3.5	2.4	2.8	3.5	2.4	2.9	3.5

Values within the group of all subjects are presented as the 25th-, 50th- and 75th-percentiles from the measurements of break up time (BUT) of the tear film in the eyes, peak nasal inspiratory flow (PNIF), forced vital capacity (FVC), and forced expiratory volume in one second (FEV_1_) measured during spirometry

The results from the linear mixed model analysis, performed for the nasal and eye symptoms as well as the BUT and PNIF measurements, are shown in Table 6.

**Table 6:** Linear mixed model results for self-assessed nasal and eye symptoms, and measured BUT and PNIF

	All subjects		Cleaning workers		Non-cleaning workers	
	Foam	Spray	Foam	Spray	Foam	Spray
Nasal symptoms	2.4 (-0.6 to 5.4) *p* = 0.11	4.0 (1.0 to 7.1) *p* = 0.009*	3.0 (-1.2 to 7.2) *p* = 0.16	2.9 (-1.5 to 7.2) *p* = 0.19	2.3 (-1.7 to 6.3) *p* = 0.25	6.4 (2.6 to 10.2) *p* = 0.002*
Eye symptoms	-0.1 (-1.5 to 1.4) *p* = 0.90	0.6 (-1.0 to 2.1) *p* = 0.47	-1.6 (-3.8 to 0.5) *p* = 0.14	-0.8 (-3.0 to 1.4) *p* = 0.48	1.9 (0.2 to 3.5) *p* = 0.03*	2.3 (0.7 to 4.0) *p* = 0.006*
BUT (sec)	-3.3 (-5.6 to -0.9) *p* = 0.007*	-2.7 (-5.7 to 0.4) *p* = 0.08	-0.9 (-2.6 to 0.8) *p* = 0.28	-0.2 (-1.9 to 1.5) *p* = 0.79	-3.9 (-8.1 to 0.3) *p* = 0.07	-6.4 (-12.5 to -0.4) *p* = 0.04*
PNIF (L/min)	-4.7 (-11.7 to 2.3) *p* = 0.19	-10.9 (-19.1 to -2.7) *p* = 0.01*	-6.7 (-16.6 to 3.3) *p* = 0.19	-12.6 (-24.9 to -0.4) *p* = 0.04*	-1.4 (-11.3 to 8.5) *p* = 0.77	-10.7 (-22.3 to 0.8) *p* = 0.07

Values are the estimated difference of the *foam* and *spray* exposures compared to the *water* exposure, the 95% confidence interval (in brackets) and the significance (p) derived from the linear mixed model. Statistical significance at a level of *p* < 0.05 is indicated with a star (*). Results from the self-assessed eye and nose symptoms and measurements of the break up time (BUT) of the tear film in the eyes and the peak nasal inspiratory flow (PNIF) through the nose are also shown

A significant increase in self-assessed nasal symptoms was found during *spray* compared to *water* for the whole group (*p* = 0.009). When the two groups were studied separately, this increase was observed only in the non-cleaning workers, suggesting that with regards to self-assessed nasal symptoms, non-cleaning workers were more sensitive to spray use than cleaning workers.

No significant differences in self-assessed eye symptoms could be observed for all subjects for either spray or foam use compared to water. When comparing the two groups, an increase was again observed only in the non-cleaning workers.

For the whole group, a significant decrease of the BUT value was observed during *foam* (*p* = 0.007) and a non-significant decrease during *spray* (*p* = 0.08). Again, this decrease was observed only in the group of non-cleaning workers, not in the group of cleaning workers.

**Fig. 5:**
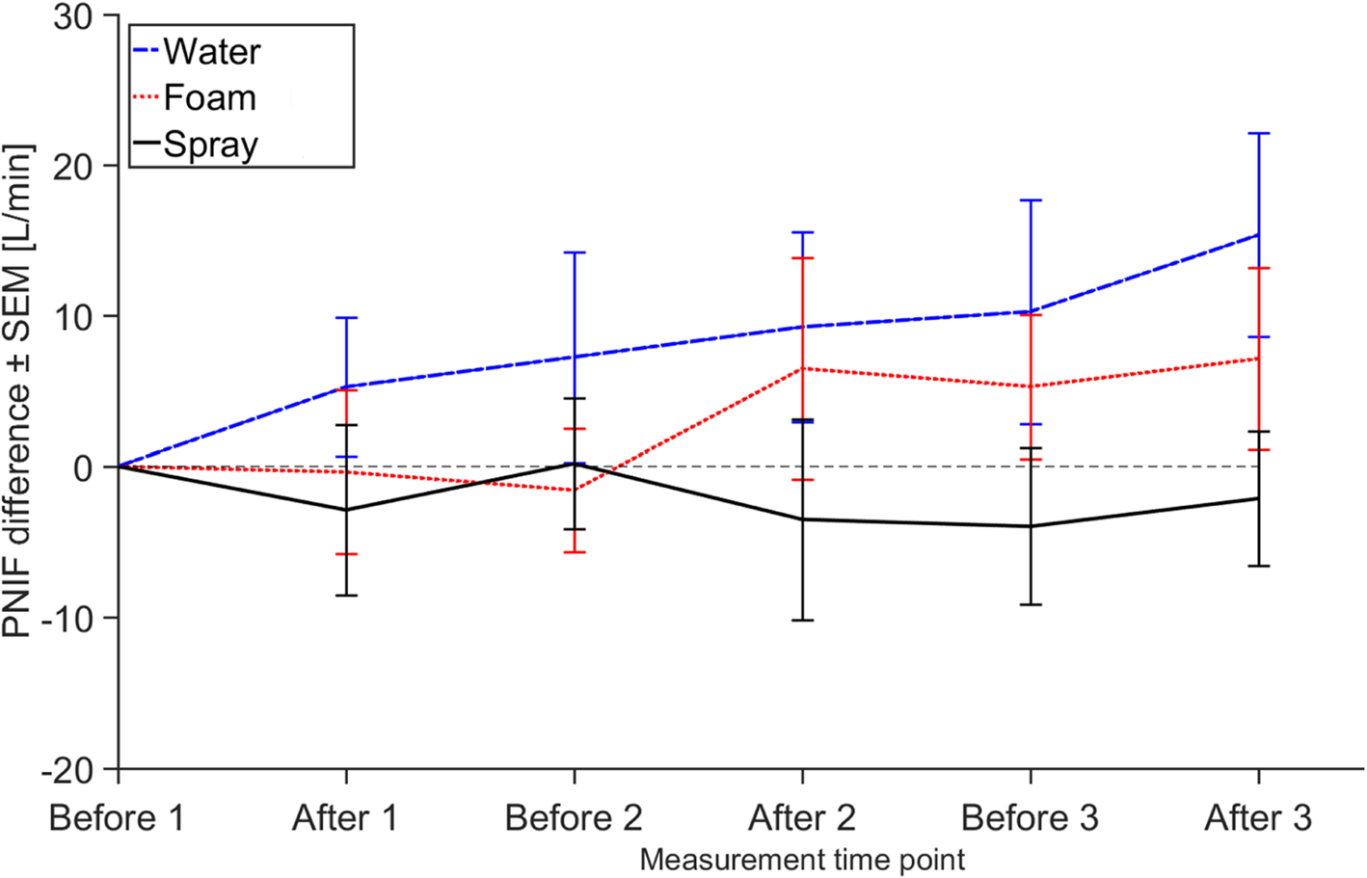
Peak Nasal Inspiratory Flow (PNIF) difference for the three different cleaning scenarios. The average difference in PNIF value at each measurement time point compared to the first measurement in the morning, before the first half-hour cleaning exposure (individually normalized), for all 19 subjects, is shown. The error bars show the standard error of mean (SEM)

The effects measured by PNIF are significant (*p* = 0.01) when looking at the whole group of subjects, visualized in Fig. 5 as the average difference in PNIF value at each measurement time point compared to the first measurement in the morning. Contrary to the above mentioned medical assessments, the only significant effect found in the group of cleaning workers was a decrease in PNIF during spray use (*p* = 0.04). Among the non-cleaning workers there was also a decrease in PNIF, however not statistically significant (*p* = 0.07).

Physical examinations of the eyes, nose and throat and auscultation of the lungs did not reveal any significant differences between the different exposure scenarios.

The measured values of the different biomarkers in blood and nasal lavage are shown in Table 7. Generally, the Wilcoxon signed rank test revealed no significant changes in the levels of biomarkers between the different exposure scenarios, except for total leukocytes (*p* = 0.018), lymphocytes (*p* = 0.013) and monocytes (*p* = 0.030), which show significant decreases during *spray* compared to *water*.

**Table 7:** Biomarkers in blood and nasal lavage during the three different exposure scenarios

	Time point	Water			Foam			Spray		
		Percentile								
		25^th^	50^th^	75^th^	25^th^	50^th^	75^th^	25^th^	50^th^	75^th^
Blood Hb (g/L)	Before 1	122	133	135	124	129	137	121	128	136
	After 3	121	128	136	118	125	135	120	124	132
	Day after	122	127	134	121	125	139	122	128	139
Blood cells Leukocytes (10^9^/L)	Before 1	5.3	5.6	6.6	5.6	6.1	7.3	5.0	6.1	7.5
	After 3	5.8	6.7	9.2	6.3	7.4	9.2	5.6	6.7	8.8
	Day after	4.8	5.5	6.7	5.2	6.1	7.5	4.9	5.7	7.2
Blood cells Neutrophils (10^9^/L)	Before 1	2.3	3.3	4.0	2.7	3.6	4.4	2.7	3.5	4.2
	After 3	3.0	4.0	5.1	3.8	4.5	5.2	3.3	4.1	4.9
	Day after	2.1	3.0	3.9	2.4	3.5	4.2	2.3	3.0	3.9
Blood cells Eosinophils (10^9^/L)	Before 1	0.1	0.1	0.2	0.1	0.1	0.3	0.1	0.2	0.2
	After 3	0.1	0.1	0.3	0.1	0.1	0.2	0.1	0.2	0.2
	Day after	0.1	0.1	0.2	0.1	0.1	0.2	0.1	0.1	0.2
Blood cells Basophils (10^9^/L)	Before 1	0	0	0	0	0	0.1	0	0	0.1
	After 3	0	0	0.1	0	0	0.1	0	0.1	0.1
	Day after	0	0	0.1	0	0	0.1	0	0.1	0.1
Blood cells Lymphocytes (10^9^/L)	Before 1	1.7	2.0	2.3	1.5	2.0	2.8	1.5	2.0	2.4
	After 3	1.9	2.1	2.7	1.7	2.1	2.5	1.5	2.0	2.6
	Day after	1.6	2.0	2.4	1.3	1.9	2.6	1.7	2.0	2.6
Blood cells Monocytes (10^9^/L)	Before 1	0.4	0.5	0.5	0.4	0.5	0.6	0.4	0.5	0.6
	After 3	0.4	0.5	0.7	0.4	0.6	0.6	0.4	0.5	0.6
	Day after	0.4	0.5	0.6	0.4	0.4	0.6	0.4	0.5	0.6
Blood CRP (mg/L)	Before 1	0	0.6	2.5	0	0	3.7	0	1.1	2.9
	After 3	0	0	2.5	0	0	3.6	0	1.1	2.8
	Day after	0	0.7	3.0	0	0	3.6	0	0.9	2.7
Blood IL-6 (pg/mL)	Before 1	0	0	0	0	0	0	0	0	0
	After 3	0	0	0	0	0	0	0	0	0
	Day after	0	0	0	0	0	0	0	0	0
Blood IL-8 (pg/mL)	Before 1	3.7	5.2	6.2	4.2	5.2	6.3	4.1	5.3	6.2
	After 3	3.5	4.6	5.6	3.3	4.6	5.6	3.4	4.2	5.6
	Day after	4.0	5.5	6.9	3.9	5.9	6.9	4.3	5.3	7.4
NL IL-6 (pg/mL)	Before 1	0	0	0.8	0	0	0.3	0	0.2	0.4
	After 3	0	0	0.4	0	0	0.2	0	0.1	0.6
	Day after	0	0	0.8	0	0.2	0.7	0	0.2	0.6
NL IL-8 (pg/mL)	Before 1	18	31	75	16	35	57	17	42	75
	After 3	14	21	38	15	24	32	16	29	60
	Day after	31	45	61	18	53	72	30	52	125

Values are presented as the 25th-, 50th- and 75th-percentiles from the measurements of biomarkers in blood and nasal lavage (NL)

The average pulse for the whole group during cleaning in the chamber was 132 ± 13% of the average resting pulse in the morning.

#### Physical workload

Both during cleaning with *spray* and *water*, the head posture varied between a considerable backward extension and a substantial forward flexion (Table 8). Though statistically significant, the difference between the methods was only 3° (somewhat more backward for *water*). Upper arm elevation (90^th^ percentile) was high for both methods and higher (6°) for *water*. Upper arm and wrist velocity were high for both methods (about 80°/s and 30°/s respectively), and there were no statistically significant differences between them. In both methods muscular recovery time was only a few % time, and the muscular load was high for both shoulder and forearm muscles. For the latter, the load was higher during *spray* than during *water*.

**Table 8:** Physical workload during bathroom cleaning for the eleven cleaning workers during *spray* and *water* exposure

	percentile	Water	Spray	*p*-value
		Mean (SD)	Mean (SD)	
Postures (°)				
*Head*				
Inclination	10^th^	-22 (7)	-19 (6)	0.01*
	50^th^	23 (19)	21 (10)	0.03*
	90^th^	59 (15)	56 (13)	0.09
*Back*				
Inclination	50^th^	18 (8)	17 (7)	0.06
*Upper arm, right*				
Elevation	50^th^	37 (9)	35 (7)	0.07
	90^th^	85 (18)	79 (17)	0.03*
*Wrist, right*				
Flexion	10^th^	-55 (9)	-52 (6)	0.03*
	50^th^	-21 (8)	-22 (8)	0.25
	90^th^	8 (12)	7 (11)	0.08
Velocity (°/s)				
*Upper arm, right*	50^th^	85 (36)	82 (32)	0.25
*Wrist, right*	50^th^	32 (10)	33 (9)	0.72
Muscular load				
*Shoulder, right*				
Recovery (% time)		4 (5)	3 (2)	0.39
Activity (%MVE)	90^th^	27 (11)	28 (11)	0.86
*Forearm, right*				
Recovery (% time)		3 (4)	1 (2)	0.08
Activity (%MVE)	90^th^	32 (7)	37 (9)	0.01*

Values are mean and standard deviation (SD) of postures and movements of the head, back, and right upper arm and wrist as well as the muscular load in the shoulder and forearm muscles. Differences between *spray* and *water* were calculated using Wilcoxon signed rank tests. Statistical significance at a level of *p* < 0.05 is indicated with a star (*)

## Discussion

A survey and a human chamber exposure study was used to investigate how often cleaning sprays are used and what exposure this generates, as well as the acute health effects of such exposure. The survey examined the use of cleaning sprays by professional cleaning workers and their symptoms. Based on the observations from the survey, a controlled human chamber exposure study was conducted to quantify the exposure of airborne particles and VOCs generated by three different cleaning methods and to correlate this exposure to measured health effects using cleaning products not containing bleach, chlorine, or ammonia. The exposure levels and effects during application with spray and with foam dispensers, and with pre-moistened microfiber cloths, were compared.

The particle and VOC measurements in the chamber study showed a significant increase during spray use of both particles and VOCs, similar to the bathroom cleaning VOC measurements carried out by Bello et al. [37]. In addition, a change from a spraying to a foaming nozzle resulted in a 7 times lower concentration of particles 0.5–20 µm and a 2.5 times lower gas phase VOC concentration. Changing from spray to foam application can hence drastically decrease the generation of airborne droplets that evaporate VOC and later form smaller and solid airborne particles. The reduction of VOCs when changing from spray to foam application is less, as the amount of VOC-evaporating liquid applied on the different cleaning surfaces is almost unchanged. A six-fold increase in total PM_10_ particle mass concentration measured in the breathing zone was seen during spray use compared to water use, verifying that cleaning workers are exposed to aerosols during spray use. No PM_10_ increase could however be seen when comparing foam to water. Between the groups of cleaning workers and non-cleaning workers, no significant difference in the aerosol exposure (either particle or VOCs) was seen.

Of the acute effects that were assessed in the chamber study, the nose was the most affected by spray use. In the absence of aerosol exposure, the nasal obstruction is expected to decrease (increased patency, increased PNIF value) throughout the day [38] as can be seen for *water* in our study (see Fig. 5). An increased PNIF value throughout the day was not observed in *spray*, thus suggesting a nasal obstruction. The linear mixed model analysis resulted in a -10.9 L/min significant difference (*p* = 0.01) between *water* and *spray* for the total group of all 19 subjects. This value corresponds to an almost 10% decrease from the measured morning PNIF values. When the two groups are studied separately, the effects are similar: -12.6 l/min and -10.7 l/min for cleaning workers and non-cleaning workers, respectively. For the cleaning workers the change is significant (*p* = 0.04) while it is not for the non-cleaning workers (*p* = 0.07). Whether this is just an effect of the low number of participants in the non-cleaning worker group (*N* = 8), where an odd value for one subject can affect the outcome of the whole group, cannot be answered in this study. Important to note that there was no significant difference in the aerosol exposure (either particle or VOCs) between the group of cleaning workers and non-cleaning workers in any of the exposure scenarios. During *foam*, there was a -4.7 L/min (non-significant) difference in PNIF values compared to *water*. These results indicate a dose–response relationship, with a higher exposure (generated by spray use) resulting in more nasal effects (both self-reported symptoms and PNIF measurements) than a lower exposure (generated by foam use). There was no statistically significant difference between the PNIF measurements when comparing scenario *spray* and *foam*, and conclusions regarding foam use and the effects thereof based on our relatively small number of 19 subjects thus have to be made carefully.

The PNIF results are consistent with the significant increase in self-assessed nasal symptoms during *spray* compared to *water* for the group of all subjects. There is, however, a difference in the self-assessed symptoms between cleaning workers and non-cleaning workers: even though the subjective measure of the self-assessed symptoms does not show a significant increase in nasal symptoms for the cleaning workers during spray use, the objective PNIF measurements suggests an effect. It was not possible from this study to assess if this is due to that cleaning workers might either have grown more accustomed to cleaning spray exposure through their professional work or if those who were affected by cleaning sprays have proceeded to another occupations (healthy workers effect). Ottaviano and Fokkens [39] argues that PNIF measurements should be used regularly in every outpatient clinic that treats patients with nasal obstruction. In our study, PNIF measurements offered a method to objectively detect nasal effects even when the subjects did not experience any symptoms, suggesting that it might be useful in occupational environments.

A slight decrease in BUT values could be seen for the group of all subjects (significant for *foam*, *p* = 0.007, and non-significant for *spray*, *p* = 0.08, compared to *water*), while no increases in eye symptoms were recorded. These results agree with a previous study by Nielsen et al. [3] in which it was found that the use of sprays was associated with a higher risk of nasal symptoms than eye symptoms. Furthermore, as with the PNIF measurements, non-cleaning workers showed a larger decrease in BUT values (*p* = 0.07 for foam and *p* = 0.04 for spray) and experienced slightly more eye symptoms (significant during both foam and spray use) than the cleaning workers (non-significant for all scenario-effect combinations).

One important aspect is that in this study, contrary to most previous studies [9–13], none of our cleaning products contained bleach, chlorine, or ammonia. Gonzales et al. [40] showed that both reported nasal symptoms during work and new-onset asthma and physician-diagnosed asthma are more prevalent among occupational groups exposed to quaternary ammonium compounds, chlorine/bleach, and glutaraldehyde. Our study shows acute effects in the absence of these chemicals. This seem to suggest that even milder products can adversely affect the health when used as sprays, which should be taken into account when assessing the risks faced by cleaning workers.

We could not observe any statistically significant change in FEV_1_ and FVC during any of our exposure scenarios for our participants who were all non-asthmatics. Studies in which cleaning workers with asthma were included [18, 19] could also not show coherent results regarding self-recorded expiratory flow measurements.

Although we found a non-significant increase in IL-6 in nasal lavage after *spray* compared to the other exposure scenarios, we found no significant evidence of an inflammatory reaction. The significant changes in the leucocytes for the *spray* exposure compared to *water* may be due to the lower levels of leucocytes in the morning pre-exposure sample for *water* than for *spray*.

A limitation of this study is that the subjects were inside the chamber three times during one day, each time cleaning the bathroom eight times over approximately 30 min. Even if this is representative of the number of rooms a hotel cleaning worker would clean during one workday and of the amount of cleaning chemicals used, the accumulated aerosol concentration in the chamber is higher than it would be in a real occupational environment, where the workers do not clean the same bathroom over and over. Moreover, the time for the physiological responses to develop was short. Nevertheless, the comparison between the different chamber exposure scenarios is realistic and relevant, with a significant decrease in both particles 0.5–20 µm and VOC gas phase concentration as well as a significant decrease in the acute effects observed (especially for PNIF) when a foaming nozzle was used instead of a spraying one.

The professional cleaning workers who participated in the survey were associated with some companies that used sprays and others that did not use sprays at all. Of the 225 participants, 77% used cleaning sprays. This proportion is comparable to what was found in the EPHECT (Exposure Patterns and Health Effects of Consumer Products in the EU) survey report [41], that Sweden has a high percentage of spray use for consumer household products compared to the EU average, with some 75% of the participants using bathroom cleaning products as sprays in their homes.

There was a good agreement between effects reported in the survey and effects observed in the experimental study. In line with what was seen during the chamber exposures, the survey showed that nasal symptoms were the most frequently reported self-experienced symptoms during spray use. Cleaning workers in our survey had an eight times higher risk of self-experienced symptoms when they used sprays compared to when they cleaned with other methods, and twice the risk when using cleaning sprays compared to never having used them, as well as a five times higher risk when the frequency of cleaning spray use increased. These results indicate a dose–response correlation between using and not using cleaning sprays as well as between the frequency of cleaning spray use and the amount of self-experienced symptoms. To our knowledge, this type of detailed survey investigating the frequency of cleaning spray use and effects thereof has only been done once before [16]. They did, however, study the use of cleaning products in homes and they found that incidence of asthma was correlated with an increased frequency of spray use. Other earlier studies have shown that using sprays increases the risk of both eye, nasal, and respiratory symptoms as well as the prevalence of asthma [3, 8, 14, 17]. In our survey, sporadic missing answers to specific symptom questions were interpreted as the worker having no such symptoms. Our observed risk of experiencing symptoms during spray use may therefore be an underestimation.

Finally, as expected, prevalence of msuculoskeletal pain was high in the survey, and the ergonomic load was high in the chamber study. In the latter, both cleaning methods tested (*spray* and *water*) yielded high loads, comparable with loads found in previous studies [21]. The muscular load on the right forearm was somewhat higher during *spray* than during *water*. One explanation for this may be the repeated handgrip when spraying. On the other hand, the arm elevation (90^th^ percentile) was lower during *spray* than during *water*. The ergonomic load during *foam* was not recorded but is expected to be similar to that during *spray*, since the foam is applied to the cloth by the same repeated handgrip as when spraying directly on surfaces*.* Thus, from an ergonomic point of view, there was no difference in the risk of developing musculoskeletal disorders between the various cleaning methods.

## Conclusions

Sprays are developed to facilitate cleaning, but this study has shown that spray use generates higher concentrations of aerosols compared to other cleaning methods, and that this results in an increase of self-experienced nasal symptoms as well as a decrease in PNIF values, even though the studied sprays were bleach, chlorine, and ammonia free. Using a foaming nozzle instead of a spraying nozzle decreases the exposure to aerosols, and thereby the nasal effects. No increased ergonomic risk is expected from such a change. Thus, if the use of cleaning products is necessary, the foaming nozzle should be considered – which would also likely lead to an improved working environment.

## Data Availability

The datasets generated and analyzed in the current study are available from the corresponding author on reasonable request.

## Abbreviations

AER: Air Exchange Rate
APS: Aerodynamic Particle Sizer
AR(1): Autoregressive
BUT: Break-up time
CAS: Chemical Abstracts Service
CI: Confidence intervals
CPC: Condensation Particle Counter
CRP: C-Reactive protein
EMG: Electromyography
EPHECT: Emissions, exposure patterns and health effects of consumer products
EU: European Union
F: Flammable
FEV_1_: Forced expiratory volume in one second
FVC: Forced vital capacity
Hb: Hemoglobin
HR: Heart rate
IL-6/8: Interleukin 6/8
MVC: Maximal voluntary contraction
MVE: Maximal voluntary electrical activity
NL: Nasal lavage
PNIF: Peak nasal inspiratory flow
RH: Relative humidity
RR: Relative risk
MSDS: Material safety data sheet
SEM: Standard error of mean
ULPA: Ultra-low penetration air filter
VAS: Visual analogue scale
VOC: Volatile organic compound
Xi: Irritant
Xn: Harmful
